# Supplementary material for: Host density drives viral, but not trypanosome, transmission in a key pollinator
Source: Proc Biol Sci. 2020 Jan 8;287(1918):20191969. doi: 10.1098/rspb.2019.1969 (PMC7003466; doi:10.1098/rspb.2019.1969)
Supplement: ESM file 1 [file rspb20191969supp1.pdf]

Host density drives viral, but not trypanosome, transmission in a key pollinator: Supplementary material

Emily J Bailes[1,2], Judit Bagi[1,3], Jake Coltman[4], Michelle T Fountain[5], Lena Wilfert[6], Mark J F Brown[1]
[1]Royal Holloway University of London, Department of Biological Sciences, Bourne Building, Egham TW20 0EX, UK
[2]University of Sheffield, Department of Molecular Biology and Biotechnology, Firth Court, Sheffield, S10 2TN
[3]Ear Institute, University College London, 332 Gray's Inn Road, London WC1X 8EE, UK
[4]Expedia Group, Angel Building, 407 St John Street, EC1V 4AD
[5]NIAB EMR, New Road, East Malling, Kent ME19 6BJ, UK
[6]Institute of Evolutionary Ecology and Conservation Genomics, University of Ulm, 89069 Ulm, Germany

This supplementary material is associated with the article found at https://doi.org/10.1098/rspb.2019.1969

Contents

Supplementary methods

i) Screening of colonies for parasites
ii) Colony sizes at the start of the field experiment
iii) Environmental conditions within the polytunnel
iv) Molecular methods, including categorisation of SBPV level
v) Preparation of SBPV inoculum
vi) Experimental timeline and setup vii) Sampling of hoverflies from within the polytunnel
viii) Packages used for statistical analyses and data visualisation
ix) Statistical analyses using metrics for drifting and a continuous measure of bee density
x) Statistical analysis of SBPV level data using a Bayesian approach

Supplementary results

i) Which flowers were present during the experiment? was there evidence of SBPV on these flowers?
ii) Is there evidence of SBPV replication in our bumblebees?
iii) Did drifting of bees between colonies influence our results?
iv) The time taken for a colony to become infected with *Crithidia*: the effect of alternative dates for colony infection in compartment F and the effect of drifting
v) The influence of density and drifting on SBPV prevalence: extra analyses

vi) The influence of density and drifting on SBPV level: extra analyses
vii) Initial and final models used to evaluate the transmission of *Crithidia* and SBPV
viii) The influence of density on SBPV level: Bayesian analyses

Supplementary references

Supplementary methods

i) Screening of colonies for parasites

Colonies were screened for the presence of the microparasites *Crithidia* spp., *Apicystis bombi,* and *Nosema* spp. by viewing faecal samples from either the queen or at least 4 workers at x400 magnification using a Nikon eclipse 50i phase microscope. Colonies were screened for the viruses ABPV, BQCV, DWV-A, DWV-B, SBPV, and SBV by RT-PCR (see section (iv); primers in Table S1) by removing 4 workers, starving them of pollen for 2 days to clear their faeces of potential contamination from pollen, then sacrificing at -80 °C. No microparasites were detected in our colonies. Viruses were detected in 69% of colonies, but SBPV was not detectable in any colony.

Table S1 - Primer pairs and RT-PCR conditions used during this study

Target	Purpose	Primer name	Primer sequence	T_a	Reference	Product size
***Bombus* Arganine Kinase**	Check cDNA quality	AK_qPCR_F	CCAGCTGGTGAGTTCATCGT	55 ºC	this study	170bp
		AK_qPCR_R	AGTTCCCTTGAGTTCACCCT			
**ABPV**	RT-PCR screen	ABPV_F5088	CYATGGACACACCCTATGTG	55 ºC	[1]	1034bp
		ABPV_R6122	CGCCATTTTGGTACTTCTCC			
**BQCV**	RT-PCR screen	BQCV_F4119	TCCYCCAGTTCAACCATCTA	60 ºC	[1]	1257bp
		BQCV_R5376	AACGTTGCCTAGRTTCGTCA			
**DWV-A**	RT-PCR screen	DWV-F7993	AACTGGCGAYCATACTCAGC	60 ºC	[2]	644bp
		DWV-8577R	WCCAGGCACMCCACATACAG			
**DWV-B**	RT-PCR screen	152 (F)	CTGTAGTTAAGCGGTTATTAGAA	55 ºC	[3]	1428bp
		154 (R)	CTGAAGTACTAATCTCTGAG			
**SBV**	RT-PCR screen	SBV-VP1b-F	GCACGTTTAATTGGGGATCA	55 ºC	[4]	693bp
		SBV-VP1b-R	CAGGTTGTCCCTTACCTCCA			
**SBPV**	RT-PCR screen	SBPV_9_774F	GAGATGGATMGRCCTGAAGG	55 ºC	[1]	915bp
		SBPV_9_1689R	CATGAGCCCAKGARTGTGAA			
**SBPV**	qRT-PCR	SPV-F3177	GCGCTTTAGTTCAATTGCC	53 ºC	[1]	226bp
		SPV-B3363	ATTATAGGACGTGAAAATATAC			

Target	Purpose	Primer name	Primer sequence	T_a	Reference	Product size
**SBPV**	negative strand specific cDNA synthesis	adapter-SBPV_F997	cttggttagctgtgttgcagttgGATGCTAACTGACCGATGG	na	this study	na
**SBPV**	synthesis of qPCR standard	SBPV_1547R	CAAACAGGCTAACATCCAAAC	55 ºC	this study	574 bp with adapter-SBPV_F997
**SBPV**	qRT-PCR (negative strand detection)	adapter	cttggttagctgtgttgcagttg	61 ºC	(Ryabov et al., 2014), this study	130bp
		SBPV_qR2b	TGCACCCAACTCTGTGGAAACT			

ii) Colony sizes at the start of the experiment

At the start of the experiment colonies were sampled for virus detection (20% of the colony or minimum of 3 bees) and then culled to as close to 16 workers as possible (Table S2). In some cases the colony size at the start of the experiment is slightly less than 16 workers because the colony was small in size before sampling.

Table S2 – Colony sizes at the start of the field experiment after culling

Colony	Compartment	Treatment	Initial size before culling (workers)	Colony size after sacrifice and sampling
20	A	RECIPIENT	33	16
8	A	SBPV	28	16
9	A	CRITHIDIA	32	16
4	B	RECIPIENT	27	16
5	B	RECIPIENT	46	16
12	B	RECIPIENT	16	13
7	B	CRITHIDIA	40	16
32	B	SBPV	35	16
37	B	RECIPIENT	17	13
10	C	RECIPIENT	38	16
11	C	SBPV	22	16
6	C	CRITHIDIA	22	16
16	D	RECIPIENT	25	16
29	D	CRITHIDIA	21	16

13	D	RECIPIENT	15	12
14	D	RECIPIENT	19	15
15	D	RECIPIENT	33	16
30	D	SBPV	23	16
28	E	CRITHIDIA	38	16
19	E	RECIPIENT	22	16
35	E	SBPV	23	16
22	F	CRITHIDIA	29	16
23	F	RECIPIENT	14	11
24	F	SBPV	48	16
17	F	RECIPIENT	16	13
26	F	RECIPIENT	42	16
3	F	RECIPIENT	13	10

iii) Environmental conditions within the polytunnel

The minimum and maximum temperature of each compartment was recorded daily throughout the period that colonies were sampled. The minimum (night) temperature ranged from 7 – 18 ºC and was on average 13 ºC. The maximum (day) temperature ranged from 29 – 44 ºC and was on average 36 ºC.

iv) Molecular methods

RNA extraction

To screen individual workers for SBPV, bees were bisected lengthwise on dry ice and their eyes removed. Bees were homogenised in 500 µl of TRI reagent in a tissue lyser II (Qiagen) at 30 Hz for 2 min followed by 20 Hz for 2 min. A further 200 µl of TRI reagent was then added to each sample. Homogenised samples were then centrifuged for 15 minutes at 12,000 g, 4°C. Following centrifugation, 350 µl of supernatant (equivalent to ¼ bee) was processed using a Direct-zol™ RNA MiniPrep kit (Zymo Research, California, USA) following the manufacturer’s protocol, which includes an on-column DNA digestion. Samples were eluted in 30µL RNase/DNase-free water. The concentration of RNA was determined using a NanoDrop.

For the initial screening of colonies for viruses, the above protocol was carried out using a 35-40 mg subsample of pooled tissue from a single colony. Samples were homogenised in liquid nitrogen instead of using a tissue lyser II, and the entirety of the supernatant was transferred to the column following centrifugation.

Flowers were screened by washing flowers in 700 µl of TRI Reagent and then extracting RNA from the Tri-reagent wash as described above.

Total complementary DNA (cDNA) was synthesised from 800 ng of RNA with 0.2 µg random hexamers (Invitrogen) and 0.4 µl of OligodT (Primer design). RNA and primers were initially incubated at 70 ºC for 5 minutes and then additional reagents added to a final reaction volume of 20 µl containing 160 U M-MLV Reverse Transcriptase (Promega), 0.5 mM dNTP (each), and 1X concentration of M-MLV reaction. Samples were incubated at 25 ºC for 10 minutes, 37 ºC for 50 minutes, and then inactivated at 70 ºC for 15 minutes.

The housekeeping gene arginine kinase was amplified from all bee samples by RT-PCR (see text below; Figure S1) to confirm cDNA quality.

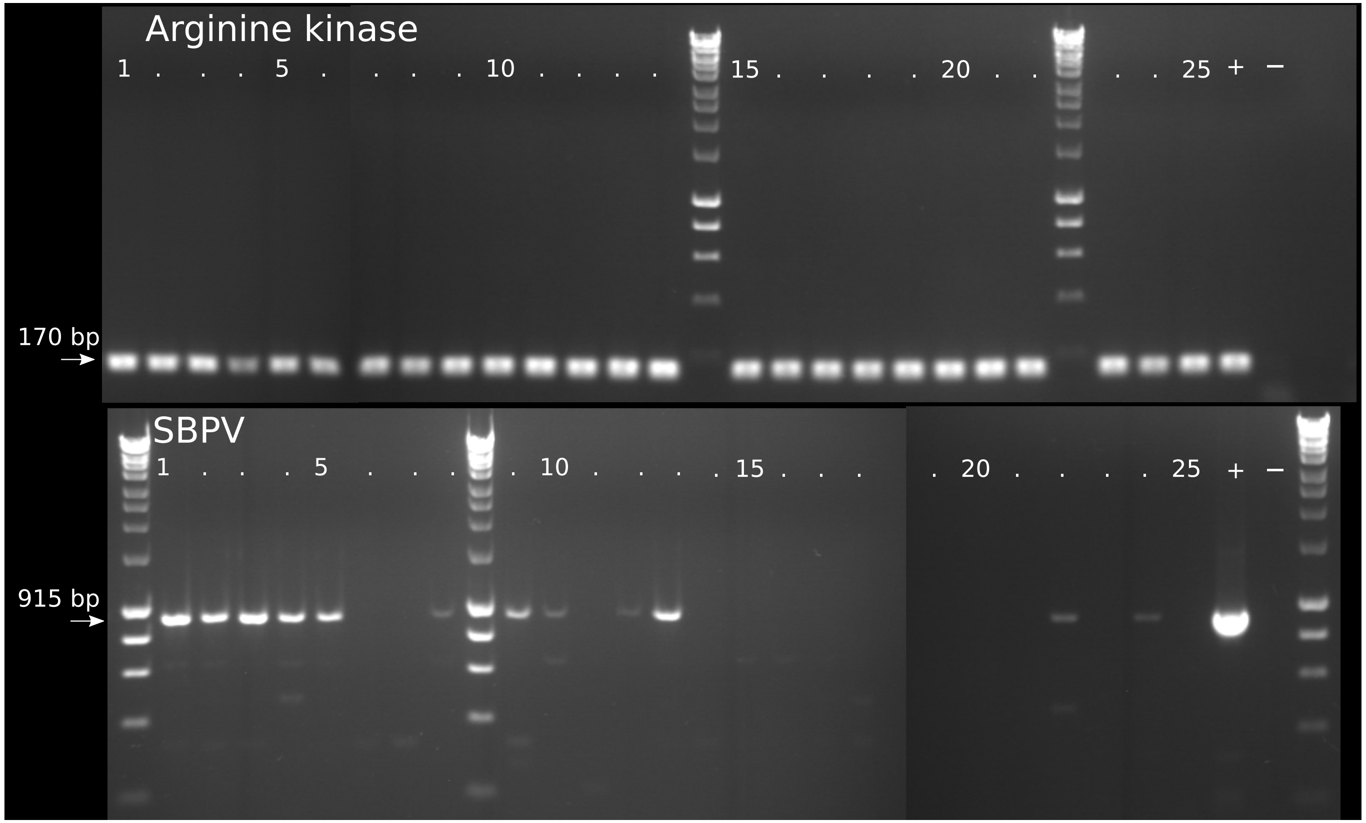

Figure S1 – Example gel showing cDNA quality. The same samples (1 – 25) are shown above for RT-PCR amplification of *Bombus terrestris* arginine kinase (170 bp fragment), and below for RT-PCR of SBPV (915 bp fragment). The 25 samples are a mixture of bees from time point 3 & 5, a positive control (cDNA from SBPV+ bumblebee) and negative control (dH_2O) are included in both images. Samples were run against a HyperLadder™ 1kb ladder (Bioline). This figure is a composite of 4 gel images. The top two images (split between samples 6 & 7) are of the same agarose gel, as are the and bottom two images (split between samples 18 & 19).

Initial screen for common bee viruses and detection of SBPV by RT-PCR

RT-PCR was used to screen samples for the presence of viral RNA. A total reaction volume of 20 µl was used, containing 0.5 U of GoTaq G2 flexi polymerase (Promega), 2.5 µl of template (0.1x cDNA), 1x reaction buffer, 2.5 mM $MgCl_2$, 0.2 µM dNTPs (each), and 0.25 µM primers (each). Samples were amplified at 95 °C for 2 minutes, followed by 37 cycles of 95 °C for 30 seconds, T_a for 30 seconds, and 72 °C for 1 minute, and a final extension step of 72°C for 5 minutes (T_a and primer sequences are given in Table S1). Positive and negative controls were included in each PCR run. PCR products were visualised under UV light on a 1.5% agarose gel stained with 0.3 µg/ml ethidium bromide.

To reduce the likelihood of false positives and to get a qualitative estimate of how much virus each sample contained, all experimental samples that tested positive for SBPV were tested a second time, following the same protocol as above but run for only 35 cycles. The band intensity was then categorised as a strength from 0 to 4, where 0 = no virus and 4 = the most virus (see "Categorisation of SBPV level").

Categorisation of SBPV level

To categorise the level of virus present in the sample, 10 µl of sample was run on an agarose gel and compared with 5 µl of Hyperladder 1kb (Bioline). The band intensity was then classified using the following rules (also see Figure S2): level 0 - no product/incorrect sized product only; level 1 - correct product visible, but band fainter or equal in intensity to 20 ng DNA (200 bp band of the ladder); level 2 - correct product visible, band intensity between 20 – 100 ng DNA; level 3 - correct product visible, band intensity greater or equal to 100 ng DNA (band oval in shape due to large amount of DNA); level 4 - correct product visible, band large, misshapen in shape, with a substantial smear on the gel due to excessive amount of DNA loaded.

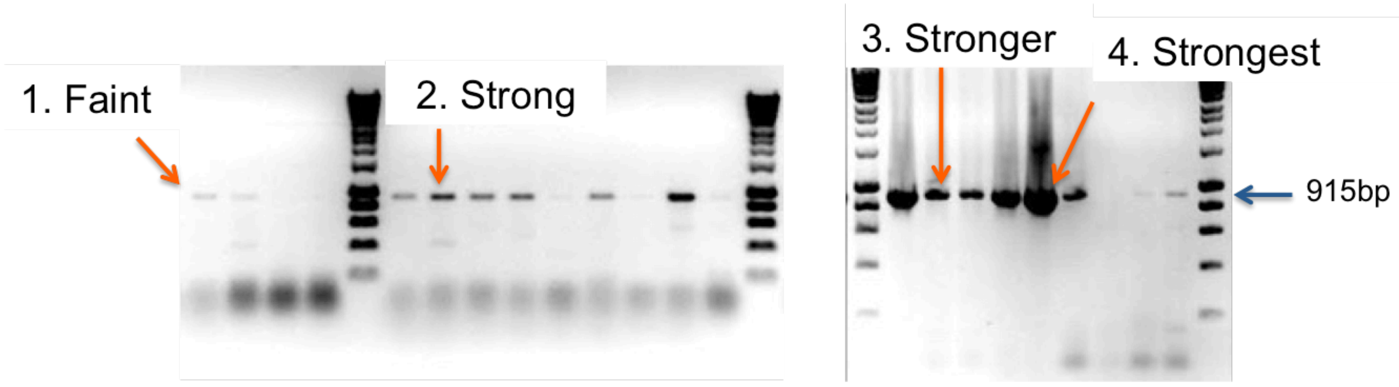

Figure S2 – Examples of products of level 1 – 4 of SBPV. The correct product is 915 bp long.

Screen of SBPV-positive samples for negative strand

To determine if there was any indication of virus replication in our colonies we tested a subset of virus-positive samples for the presence of negative stranded SBPV. This subsample was based on the intensity of band amplified during RT-PCR (ESM methods section vi). All samples that had an intensity of 3 (n = 27) and 4 (n = 12), ~half of samples with an intensity of 2 (n = 25) and ten samples with an intensity of 1, were tested.

The negative strand of SBPV was detected using the protocol of de Miranda et al. (2013; section 10.2.8.1) for Superscript III (Invitrogen). The tagged primer adapter_SBPV-997F was used for strand-specific cDNA synthesis. Excess primers were removed to reduce the chance of false positives by adding 1 μl of 1X exonuclease buffer containing 10 U exounuclease I (Thermo scientific) to 5 μl of cDNA. The reaction was incubated at 37 ºC for 30 min and then denatured at 80 ºC for 15 min.

qRT-PCR was used to detect samples positive for the negative strand of SBPV. Triplicate reactions were carried out using a Roche LightCycler® 480 II, with LightCycler® 480 SYBR Green I (Roche) mastermix with a 10 μl final reaction volume, 0.5 μM of the adapter and virus-specific primer SBPV_qR2b and 2 μl of template (0.2x cDNA). A standard curve ranging from 3.4 x 10^5 to 3.4 x 10^0 genome equivalents of SBPV was contained in each reaction run. The standard was generated from a PCR fragment amplified using the primers adapter_SBPV-997F and SBPV-1547R (as per RT-PCR conditions above) on a sample positive for negative strand RNA (individual

c24b35) then purified using a Wizard® SV Gel and PCR Clean-up System (Promega) diluted appropriately. Thermocycler conditions for qRT-PCR were as follows: 5 min at 95°C, followed by 45 cycles of 10s at 95°C, 10s at 61°C and 15s at 72°C (read). Following PCR, DNA was denatured for 5s at 95 °C and cooled to 65 °C for 1 min. A melting profile was generated from 65 to 97 °C (0.11°C per second increments) to rule out false positives. A no-template control of water was included in each reaction run. Quantification was based on the standard curve calculated on the same run. The primer pair efficiency was 93%.

For samples that amplified a product of the correct melting temperature in at least two of the three replicates, we re-synthesized cDNA from the same RNA extraction. In parallel, an identical reaction with the exception that the primer was replaced with water was set-up. This no-primer reaction was used to test for self-priming of the cDNA, which can generate false positives. We carried out a second qRT-PCR on these two new templates, using exactly the same conditions. Samples were classed as positive if the primed reaction once again produced a product of the correct melting temperature in at least 2/3 replicates **and** the no primer reaction did not amplify the correct product, or the negative primer reaction amplified a minimum of 3 cycles later than the positive replicates of the primed reaction. As a further control for false-positives, all positive samples were run in a qRT-PCR containing primed cDNA as a template, and the reverse primer only. None of these reactions amplified a product, indicating that the exonuclease treatment had successfully removed the cDNA synthesis primer, so that it could not participate in the PCR template priming. For a subset of samples (with the greatest quantity of negative strand molecules), the adapter primer was tested alone on primed cDNA to ensure the specificity of the primer pair. There was no amplification of the correct product in these reactions.

v) Preparation of SBPV inoculum

SBPV was propagated using white-eyed pupae of *Bombus terrestris audax*. Inoculum generated by homogenising five SBPV-infected bees in insect ringer solution was kindly provided by R. Manley. This was injected into pupae between the 3rd and 4th abdominal segment using a Hamilton syringe. Pupae were then incubated at 30 ^{o}C in sealed petri dishes with damp filter paper to maintain a high level of humidity. After 5 – 7 days of incubation pupae were frozen in liquid nitrogen; pupae that died during this process were discarded.

In total, 5 pupae were ground in liquid nitrogen and suspended in 5 ml of cold 0.5M KPBS (pH 8.0). The extract was then filtered through cheese cloth and clarified by centrifugation at 8,000g for 15 minutes at 4 °C. The supernatant was transferred to a fresh tube for storage at -80 °C. The RNA of a subsample of 10 µl of this extract was then extracted as described in the main text except that a tissue lyser was not used, and the entire supernatant of Tri-reagent was applied to the column. The inoculum was screened for the most prevalent viruses in the UK by RT-PCR (viruses ABPV, BQCV, DWV-A, DWV-B, SBPV, and SBV). The inoculum was negative for all viruses screened for except SBPV. The inoculum was clarified further by a centrifugation at 8,000g for 15 minutes at 4 °C. The virus was then pelleted from the supernatant by centrifugation at 75,000g for 3h at 4°C. The viral pellet was re-suspended in 140 µl of 0.5M KPBS (pH 8.0) and stored at -80 ºC.

Following RNA extraction and cDNA synthesis from a subsample of the final inoculum, the viral titre was quantified by qRT-PCR as described for SBPV negative strand, but using primers SPV-F3177 & SPV-B3363 (Table S1) and a standard curve ranging from 2×10^8 to 2×10^2 genome equivalents of SBPV and an annealing temperature of 53 °C. Quantification was based on the standard curve calculated on the same run. The final inoculum before dilution for use in experiments was estimated to be 1.8 x 10^9 copies SBPV per µl.

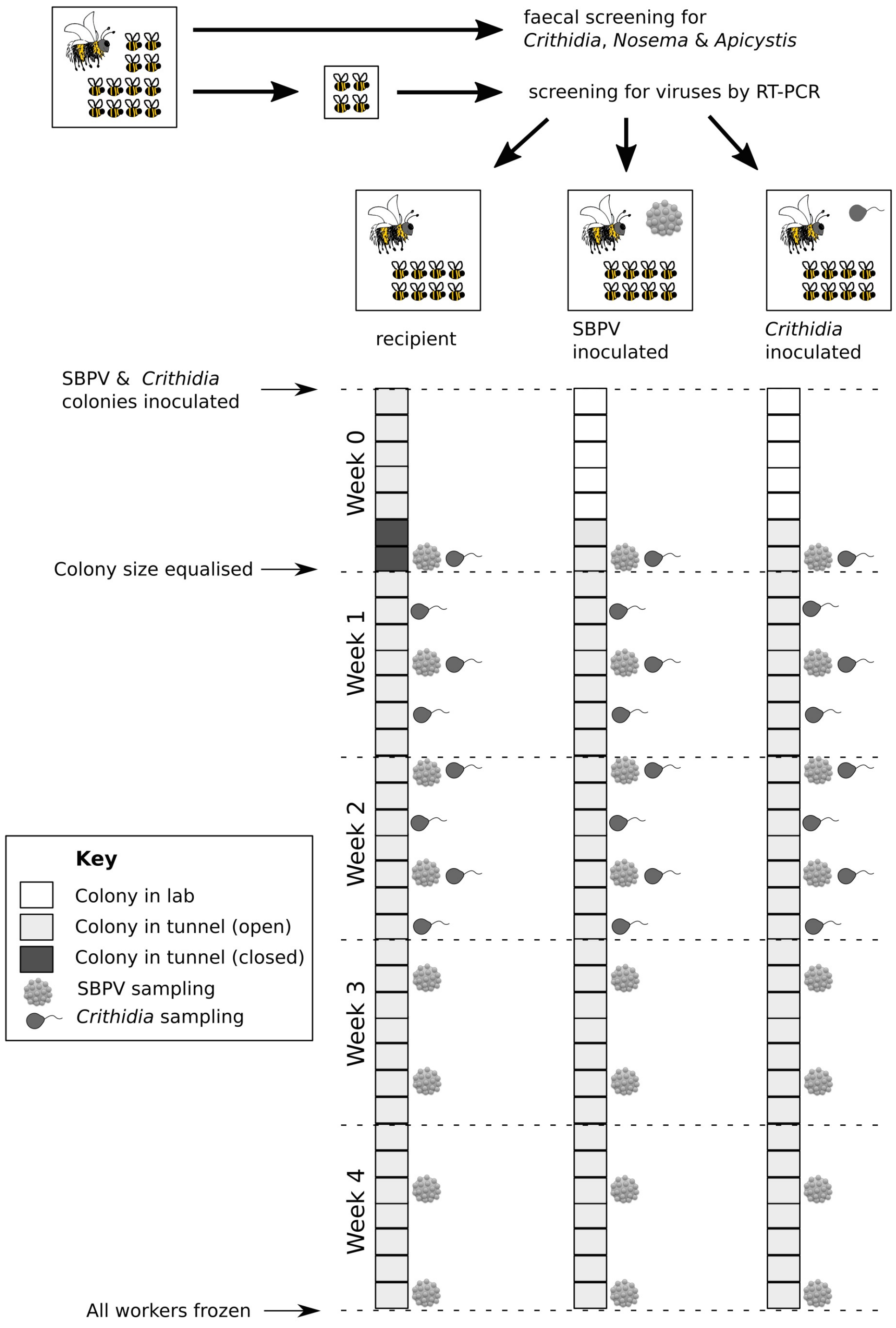

vi) Experimental timeline and setup

The experimental timeline for the experiment is shown in Figure S3. Workers with deformed wings and newly emerged male bumblebees were removed throughout the experiment. Colonies were kept at ~27 °C and 40 % humidity in a dark room and fed 44 % w/w sugar solution and irradiated pollen (Biobest) *ad libitum* in the lab. Once in the field, colonies were fed ~50 ml of 27 % w/w sugar every other day, or 22 % w/w on exceptionally hot days.

Figure S3 – Schematic of experimental timeline. Colonies were screened for common protozoan parasites and viruses before the start of the experiment. Those free of SBPV and protozoan parasites were randomly assigned to a treatment group. The filled squares represent every day after the inoculation of the donor colonies. White = colony in the lab, mid-grey = colony in the field with access to flowers, dark-grey = colony in the field but access to flowers was closed. The timepoints at which colonies were sampled for *Crithidia* (faecal samples) or SBPV (bee samples) are indicated by symbols of these parasites to the right of the day they were sampled. Each colony was sampled for *Crithidia* until at least one worker with an infection was identified

vii) Sampling of hoverflies from within the polytunnel

Over the duration of the experiment, hoverflies (Syrphidae) emerged within our tunnels. The hoverflies were presumably from eggs that had been laid in the polytunnels before the plants were in flower. To test whether these hoverflies could be a source of contamination to our experiment, we collected 3-5 hoverflies from each corridor between compartments (n = 15 total; See Figure 1 – corridors are the large white shaded areas between the compartments shaded in grey), which were contained by 1 layer of netting. RNA from these individuals was then extracted as outlined for bees, except that whole individuals were used. The head and abdomen were extracted separately. None of the samples that we tested were positive for SBPV, as determined by qRT-PCR.

viii) Packages used for statistical analyses and data visualisation

Data were plotted using ggplot2 [version 3.1.0; ,5] and sjPlot [version 2.6.1; ,6], figures were made using Inkscape version 0.91 (www.inkscape.com).

Log-likelihood ratio tests were used to calculate p-values using nested models, with either the drop1() or anova() functions in the base package of R [version 3.4.1; ,7]. Treatment contrasts were used for the factors 'treatment' (inoculation group: un-inoculated/Crithidia-inoculated/SBPV-inoculated) and 'density' (low/high). The reference group is the first listed in each case. **Multicollinearity** of all final models was examined using the vif function from the 'rms' [version 5.1-2; ,8] or 'car' [version 3.0-2; ,9] packages.

We used a **linear mixed model** to test if bumblebee nesting density was significantly associated with the response variables: flowers/compartment, flowers available/bee, and bumblebee visitation rate. This was run using the lmer function in the package 'lme4' v1.1-18-1 [12]. The assumptions of normally distributed residuals and homogeneity of variance we checked by examining quantile-quantile plots of the model residuals and a plot of the residuals against fitted value.

We used a **cox proportional hazard model** to test if bee density affects the time taken for a colony to become infected with *Crithidia*. This was run using the package 'coxme' v2.2-10. The assumption of proportional hazards was tested by using the cox.zph function and

refitting the final model using the coxph function with compartment fitted as a frailty term, using the 'survival' package v2.42-3 [10,11]. Initial and final models are given in Tables S5-8.

We used a **logistic regression model** with a logit link function to determine if bee density affected the prevalence of SBPV within a colony. This model was fitted using a glmer model from the binomial family with a logit link function in the package 'lme4' v1.1-18-1 [12]. A **cumulative link mixed model** was used to determine if the level of SBPV infection detected within a worker was affected by bee density. This model was run using the clmm function in the package 'ordinal' v 2018.8-25 [13] with a flexible threshold. Models were checked for the assumption of proportional odds by refitting the final model using clmm2. As this model can only deal with one random factor, colony was removed from the model, because its standard deviation was always very close to 0. Each predictor was then tested as a nominal variable to ensure that relaxing of the assumption of proportional odds did not significantly increase the model fit. Initial and final models are given in Tables S9-12.

ix) Statistical analyses using metrics for drifting and a continuous measure of bee density

To indicate if drifting was having a significant affect on our results, we re-ran our statistical analyses with the high/low density treatment replaced with the continuous predictors of drifting and bee density (flowers available per bee). To improve model convergence, these variables were standardised by subtracting the variable mean and dividing by the standard deviation. The most suitable measure of drifting to explain the models data was selected by running each measure of drifting in a model containing drifting as the predictor and any relevant random factors. The model with the lowest AICc (ie. the one best able to explain the data alone) was chosen as the relevant drifting metric for further model selection. For the model of *Crithidia* infection, 'number of bees in a colony drifting to/from the *Crithidia*-inoculated colony' was the best metric selected from 'percentage of colony drifting to/from the *Crithidia*-inoculated colony', 'number of bees in a colony drifting to/from the *Crithidia*-inoculated colony', 'percentage of colony drifting to/from other colonies', and 'number of bees drifting to/from other colonies'. For both models of SBPV detection, 'percentage of colony drifting to/from the SBPV-inoculated colony' was the best metric selected from 'percentage of colony drifting to/from the SBPV-inoculated colony', 'number of bees drifting to/from the SBPV-inoculated colony', 'percentage of colony drifting to/from other colonies', and 'number of bees drifting to/from other colonies'. Analyses were then carried out as described in the main manuscript.

x) Statistical analysis of SBPV-level data using a Bayesian approach

Overview

The conceptual model underlying the generation of data for this model is that at every time point:

1. Bees forage within the compartment and interact with each other, allowing potential virus transmission
2. A colony's level of infection is increased through some function of how often bees interact with other bees, and how infected those other bees are
3. A colony's level of infection changes due to factors such as infection spreading within the colony or immune response
4. A number of bees' level of infection is observed (through the detection of SBPV in our bee samples)

In the Bayesian model, the level of infection of each colony through time is modeled as a random walk. This random walk captures all of the changes in step 3.

Colonies interact with each other, and virus transmission occurs, through the mean of random walk steps. The mean of the update step is a function of:

- The previous period's level of infection
- The density of the compartment
- The level of infection of other colonies in the previous period

We model step 4, the bees' level of infection, using an ordinal logistic approach. The latent infection of a bee is drawn from the colony's infection distribution. This latent infection is compared to a series of latent thresholds that determine the required value for each SBPV-level (see below)

This model is used to test if there is a significant effect of bumblebee nest density on the transmission of SBPV through step 2 (interaction with other bees).

Model Assumptions in Detail

1) A colony's level of infection can be represented as a scalar real

The requirements are that:

- we can always rank colonies' level of infection
- it conceptually makes sense that a colony can be n-times as infected as another colony

2) At every time period, the latent infectedness of a colony takes a random step that is uncorrelated to other colonies in the compartment

We use this random walk step to capture a lot of different mechanics that would otherwise be extremely challenging, for example:

1. Infection spreading between bees within the colony
2. Bees naturally fighting off the infection

A result of this assumption is that the random walk steps are uncorrelated across colonies in a compartment. In practice there a number of reasons this assumption is violated, for example the weather conditions which are the same across compartments. However, they will be significantly smaller in magnitude than the primary variables included in the model.

3) The mean of the step that a colony makes in a time period will be impacted by the level of infectedness of the donor colony in the compartment.

There are two main parts to this assumption:

We assume that the only interaction between colonies is through the mean of the SBPV-level update step.

We assume that all of the interaction happens within a period, there is no lag. Given that the periods are quite long (4 days), this doesn’t seem unreasonable.

4) The only interaction between colonies is from donor to recipient colonies

This is a simplifying model that we build due to the limited amount of data we have available. The results of allowing all possible interactions are materially the same, but extremely unstable. While this assumption is not trivial it will only decrease the strength of the signal being measured, meaning the results of this model are conservative.

Notation

$I_{j,t}$: Latent SBPV-level of a colony in compartment j at time t

$d_{j,t}$: Latent SBPV-level of donor colony in compartment j at time t

$r_{i[j],t}$: Latent SBPV-level of recipient colony i in compartment j at time t

$O_{k[i[j]],t}$: Observed SBPV-level of the kth observation of colony i in compartment j at time t

$K_{i[j],t}$: Observations for colony i in compartment j at time t

R_j: Recipient colonies in compartment j

$Cutoffs$: The cutoff values

The model

The total likelihood is factorised into three components:

1. SBPV-level updates
2. Observations
3. Priors

1) SBPV-level updates

We use the Gaussian family for the random walk step. This distribution captures a number of our assumptions about the nature of the random step:

- colonies are as likely to become less infected as more infected

- colonies are more likely to make small steps than large ones

The variance of the update step is assumed the same for all colonies. However, the results of the model are robust to allowing the high and low density colonies to have different variances.

Colony interactions

Donor colonies have a random walk update centred on their mean, as we assume they are not impacted by any of the recipient colonies.

$$P(d_{j,t+1} | O_{k[j],t}) \propto N(d_{j,t},\ \sigma)$$

For the recipient colonies, we add a linear and additive term representing transmission of SBPV from the donor colony.

We parameterise the difference between the high and low density compartments into:

- α - baseline level of transmission
- β- additive transmission of SBPV present in high density compartments

These correspond to the intercept and the coefficient of a dummy variable for high density in a standard regression model.

This parameterisation was chosen over using a different coefficient for the low and high density compartments because:

- using all observations to estimate the coefficient for low density compartments improves stability of the MCMC sampling
- it allows us to directly sample from the additive transmission of SBPV, present in high density compartments
- it avoids having to compare distributions based on the very different numbers of observations between low and high density compartments

In low density compartments, the update step is:

$$P(r_{i[j],t+1} | O_{k[i[j]],t}) \propto N(r_{i[j],t} + \alpha d_{j,t},\ \sigma)$$

And in high density compartments:

$$P(r_{i[j],t+1} | O_{k[i[j]],t}) \propto N(r_{i[j],t} + (\alpha + \beta) d_{j,t},\ \sigma)$$

2) Observations

Conditional on the latent SBPV-level, $I_{i[j],t}$, each observation is a identical independently distributed draw from an ordinal logistic. In mathematical notation:

$$P(O_k \mid I_{i[j],t}) \propto \prod_{k \in K_{i[j],t}} OrdinalLogistic(I_{i[j],t}, Cutoffs)$$

This logic matches that in the ordinal regression model

3) Priors

We decompose the total prior into a number of independent factors. In general, priors were chosen to be as flexible as possible, while still leading to a well identified, numerically stable model

Starting SBPV-level

The donor colonies' starting SBPV-level had a prior of 0 with a wide variance to allow for the fact that we don't have strong prior knowledge about how much of an effect the inoculation had.

Recipient colonies' starting SBPV-level had a prior of 0 with a low variance. We didn't enforce this to be 0 to encode the fact that some recipient colonies were exposed to the donor colonies before the experiment started.

Cutoffs

The cutoffs for the different levels of the ordinal regression had an ordered Cauchy prior with a wide variance. The Cauchy distribution was chosen because it allowed us to capture our prior belief that the distances between the SBPV-levels could be very uneven

Standard deviation of random walk

An uninformative gamma prior was used for the standard deviation of the random walk, as we had no real prior knowledge. The results are robust to different non-informative priors.

Colony interactions terms

The terms for the virus transmission between colonies (α and β) were given an uninformative normal distribution.

Inference

The model was written in the probabilistic programming language, Stan, and analysed using the pyStan library in Python 3.6.

Samples were generated from 5 chains of 5000 samples each, with the first 2500 being used as a burn in. Samples were thinned such that only every fifth sample was kept.

Supplementary results

i) Which flowers were present during the experiment? was there evidence of SBPV on these flowers?

The majority of the floral units available for bees, and foraged on during the experiment, came from wildflowers present in the seedbank: *Matricaria recutita* (scented mayweed) and *Papaver rhoeas* (field poppy). *Medicago lupulina* (black medick), *Anthemis arvensis* (corn chamomile) and *Cynasunus cristatus* (Crested dog's tail) were also present in the seedbank and foraged on by bees. The natural seed bank was supplemented by flowers grown from a custom wildflower seed mix, with *Anthemis austriaca* (corn chamomile), *Leucanthemum vulgare* (Ox-eye daisy), *Trifolium pratense* (Red clover), *Trifolium hybridum* (Aslike clover) all flowering and foraged on during the experiment. We tested a small sample of the most frequently visited flower species, *M. recutita,* for SBPV by RT-PCR as described in (iv). To ensure that our experimental setup was free of any external sources of contamination we tested 5 flowers from each compartment, collected just prior to the bees being introduced. There was no evidence (amplification) of SBPV in any of these 30 samples. In contrast, there was some evidence of SBPV presence on flowers collected 13 days into the experiment (3/30 flowers from had detectable virus).

ii) Is there evidence of SBPV replication in our bumblebees?

We identified evidence of virus replication through the detection of negative stranded SBPV in a total of five inoculated colonies (12 bees total) and three non-inoculated colonies (1 bee/colony). All three non-inoculated colonies were located in compartment D. In total we detected evidence of virus replication intermediates in 0/10, 2/25, 5/27 and 8/12 samples from bees which had been identified as having a category 1,2,3 or 4 intensity band by RT-PCR.

iii) Did drifting of bees between colonies influence our results?

During the course of the experiment, drifting of workers between colonies was observed. To give an indication to whether this was having a significant affect on our results we recorded the workers that were in the non-parent nest every other night. Over the duration of the entire experiment there were between 0 and 11 'imposters' in a nest on a given night (2 on average). In terms of the percentage of workers within a colony that were imposters, values ranged from 0 – 100 % a night (16% on average).

iv) The time taken for a colony to become infected with *Crithidia*: the effect of alternative dates for colony infection in compartment F and the effect of drifting

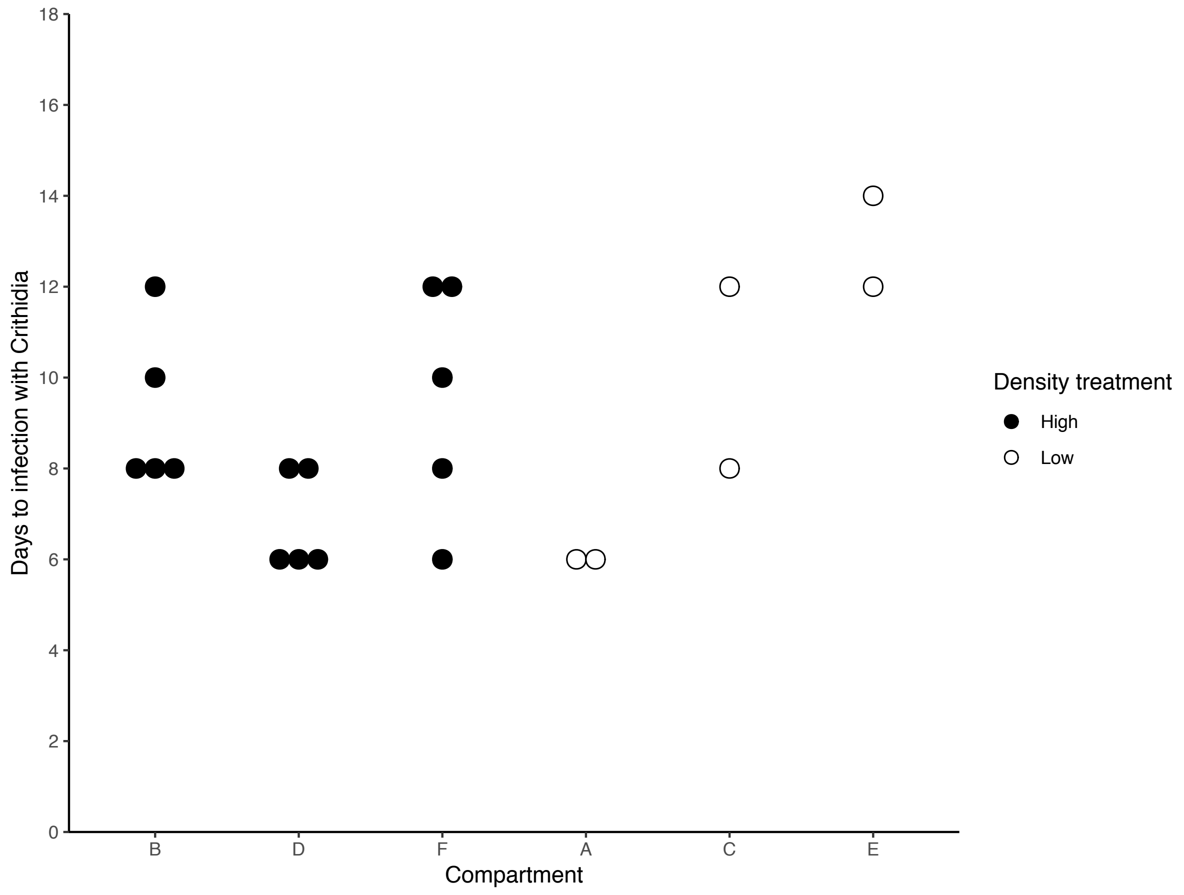

Figure S4 – The time taken to detect *Crithidia bombi* in faecal samples from each recipient and SBPV-inocualted colony, grouped by the compartment the colony was located in. Colonies in a high-density compartment are indicated by filled circles, and colonies in a low density compartment indicated by open circles. Data is for scenario 1 where the colony with an inaccurate date of infection in compartment F is infected on day 12.

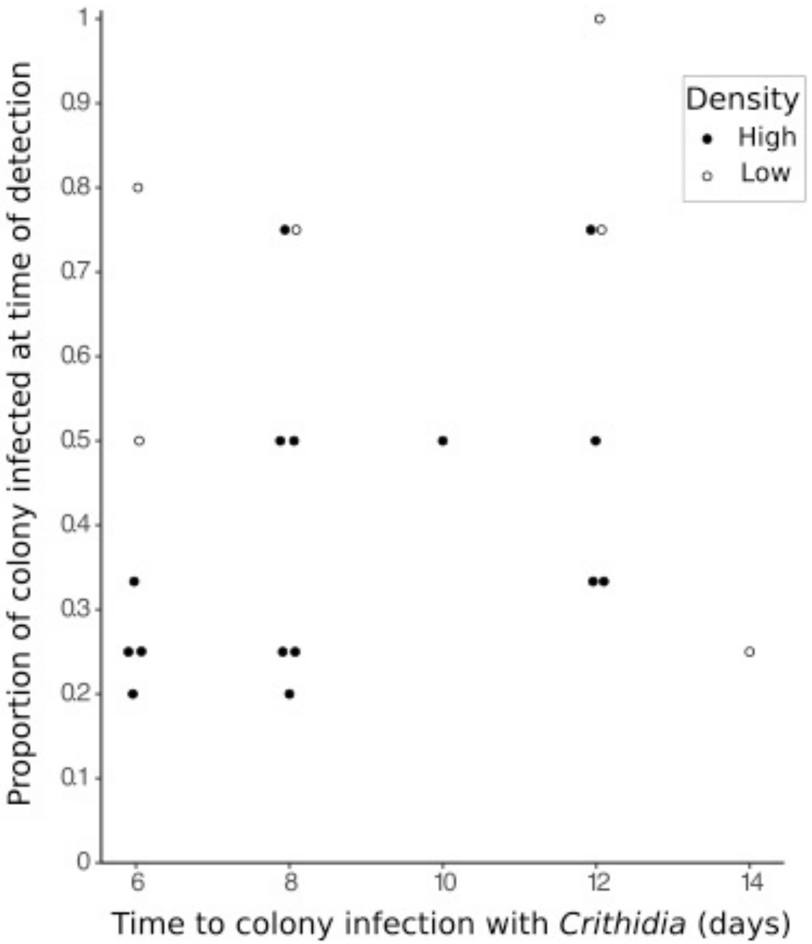

Figure S5 – the correlation between the time taken to detect *Crithidia* within a colony and the proportion of the colony infected with *Crithidia* at that time. Data are under scenario 2 (see text for details). Note, for ease of viewing, points are dodged in the x-axis; data was recorded at 2 day intervals. There is no significant relationship between these two variables (Spearman's rank correlation; rho = 0.25, p = 0.27).

During the course of sampling, colony 3 from compartment F was missed from the sampling scheme for *Crithidia* on day 10. On day 12 the colony had a detectable *Crithidia* infection, therefore it could have been detected as infected on day 10 or day 12. In the main text,

the more conservative analyses of infection at day 12 are presented (scenario 2). This analysis is robust in its conclusions regardless of whether the colony became infected on day 10 or 12 however. In scenario 1 (Figure S4; infection at day 10), the final cox-proportional hazard model contained colony treatment (hazard ratio = 0.5 when treatment = SBPV-inoculated; $X^2(1) = 0.02$, $p = 0.9$) and bee density (hazard ratio = 1.1 when density = high; $X^2(1) = 2.0$, $p = 0.16$) were both non-significant predictors of the time taken for a colony to become infected with *C. bombi*. This is essentially the same as the model under scenario 2, colony treatment (hazard ratio = 0.4 when treatment = SBPV-inoculated; $X^2(1) = 0.03$, $p = 0.9$) and bee density (hazard ratio = 1.1 when density = high; $X^2(1) = 2.5$, $p = 0.12$) were also non-significant predictors of the time taken for a colony to become infected with *C. bombi*. For scenario 2, we also analysed the correlation between time to detection of *Crithidia* within a colony and the prevalence of *Crithidia* within the colony on that date. We found no significant relationship between these two variables (Spearman's rank correlation; rho = 0.25, $p = 0.27$), suggesting that there is no systematic error over time in the estimated time to infection of a colony (Figure S5).

To give an indication of whether these results were being driven by drifting rather than the density of bees between colonies, we broke the bee density treatment down into the measured variables 'flowers available per bee' and 'drifting level'. For scenario 2 the best measure of drifting was average number of bees moving to/from the *Crithidia*-inoculated colony over the first 14 days of the experiment, whereas for scenario 1 the best measure of drifting was the proportion of bees moving to/from the *Crithidia*-inoculated colony; see methods). For scenario 2, treatment (hazard ratio = 0.5 treatment = SBPV-inoculated; $X^2(1) = 1.7$, $p = 0.19$), flowers available per bee (hazard ratio = 0.7; $X^2(1) = 1.5$, $p = 0.2$) and drifting (hazard ratio = 0.6; $X^2(1) = 1.5$, $p = 0.22$) were all non-significant predictors of the time taken for a colony to become infected with *C. bombi*. This suggests that factors other than drifting of bees between colonies and bee density are driving the time taken for *Crithidia* to be detected in a colony. Once again, the using data from scenario 1 leads to very similar estimates. Treatment (hazard ratio = 0.5 treatment = SBPV-inoculated; $X^2(1) = 1.5$, $p = 0.22$), flowers available per bee (hazard ratio = 0.7; $X^2(1) = 1.8$, $p = 0.18$) and drifting (hazard ratio = 0.6; $X^2(1) = 2.7$, $p = 0.57$) were all non-significant predictors of the time taken for a colony to become infected with *C. bombi*.

v) The influence of density and drifting on SBPV prevalence: extra analyses

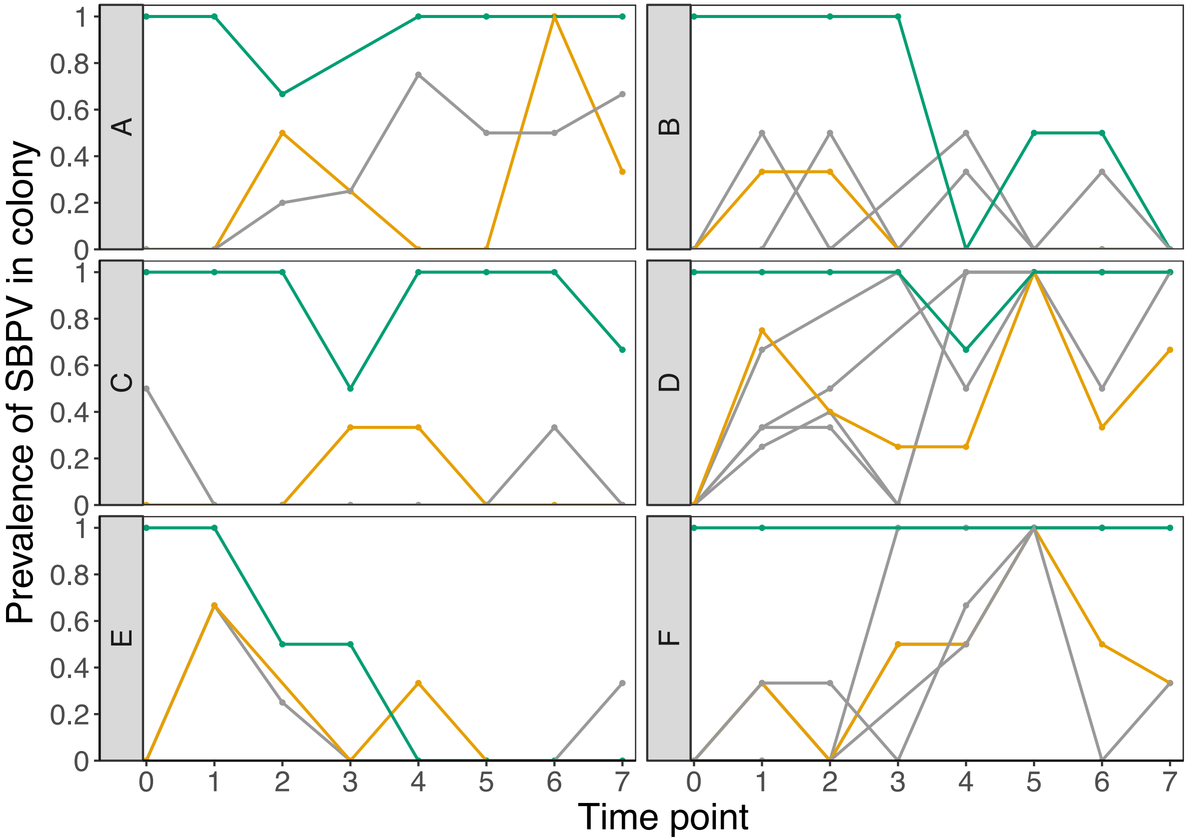

Figure S6 - Raw prevalence data of SBPV in each colony. Green = SBPV-inoculated, orange = *Crithidia*-inoculated, grey = recipient. In compartments E and B it is clear that the SBPV was not maintained to high levels and therefore these compartments are excluded from statistical analyses of SBPV prevalence and the SBPV-level in the test colonies. Time points are at 4 day intervals, where 0 = before colonies were allowed to forage together and 7 = the end of the experiment.

To investigate further how bee density may predict SBPV presence, we broke the bee density treatment down into the measured variables 'flowers available per bee' and 'drifting level' (percentage of bees in the focal colony that had drifted to or from the SBPV-

inoculated colony; see methods). The final model is very similar to that containing low/high density. In the final model, treatment:time ($X^2(1) = 8.0$, $p = 0.0046$) and bee density ($X^2(1) = 4.0$, $p = 0.045$) but not drifting ($X^2(1) = 0.25$, $p = 0.62$) were significant predictors of the likelihood of detecting virus in a bee. The model estimates are given in Table S3. These suggest that bee density is having a stronger impact on the likelihood of detecting SBPV in a bee than the drifting of bees to/from the virus-inoculated colony. When there are more flowers available per bee, the odds of virus being detected in a bee are significantly decreased. The interaction between *Crithidia*-inoculated colonies and time shows the same pattern as when density is treated as a categorical variable.

Table S3 - Model estimates for the likelihood of detecting virus in a bee including a measure of drifting and bee density treated as a continuous variable. Predictor time = the time (days) at which the bee was sampled, treatment = whether the bee was from a non-inoculated or *Crithidia*-inoculated colony, density is the average number of flowers available per a bee over the duration of the experiment, and drifting is the percentage of bees in the focal colony that had drifted to or from the SBPV-inoculated colony. Density and drifting measures were standardised across the compartments included in these analyses. Estimates for treatment are for when colonies are '*Crithidia*-inoculated' compared with the reference level of an un-inoculated colony. P-values are not reported for time or treatment alone as their interaction is statistically significant

Predictor	Estimate	SE	Odds ratio	p-value
**intercept**	-2.65939	0.51719	-	-
**time:treatment**	-0.41518	0.14667	0.66	0.005
**time**	0.65014	0.09756	1.9	-
**treatment**	0.99886	0.66632	2.7	-
**density**	-0.96453	0.35926	0.38	0.045
**drifting**	0.11583	0.22799	1.1	0.62

vi) The influence of density and drifting on SBPV level: extra analyses

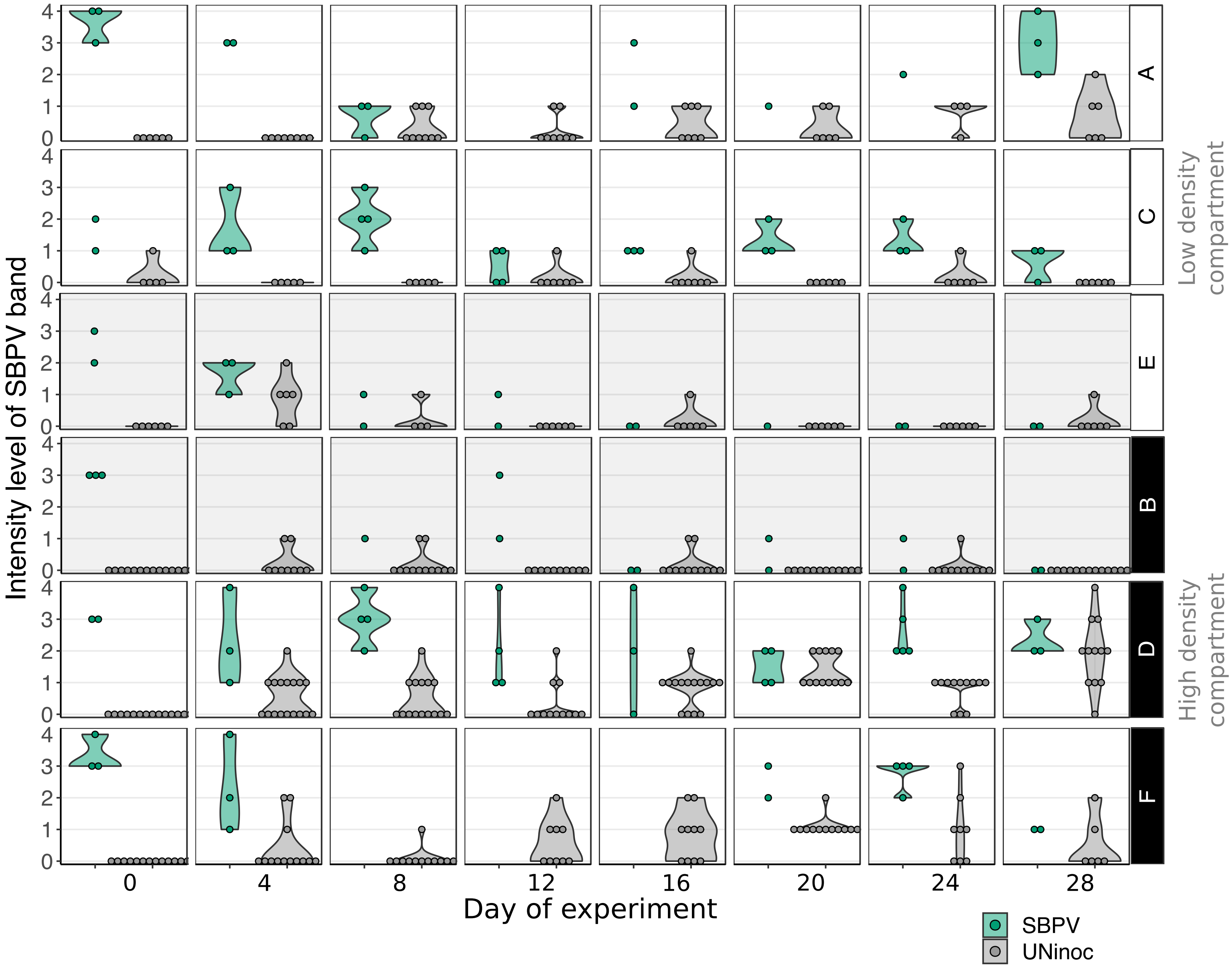

Figure S7 - The distribution of SBPV intensity level from RT-PR of samples. Samples are plotted for each compartment and day of sampling, for the SBPV-inoculated colony (green) and the un-inoculated colonies (recipient + *Crithidia*-inoculated; grey). Violin plots show the underlying density of data where n>2. Dots represent individual bees. Note that for compartment F, the SBPV-inoculated colony was not sampled on day 8 – 20 of the experiment because of poor colony growth. Compartments B & E (shaded grey background) both had SBPV donor colonies where the treatment failed.

When breaking the density treatment into the measured variables 'flowers available per bee' and 'drifting' the final model contains the interaction between time and treatment (*Crithidia*-inoculated or non-inoculated colony), 'number of flowers per bee, and 'drifting level' (percentage of bees in the focal colony that had drifted to or from the SBPV-inoculated colony), as well as the random factors compartment and colony. In this model, time:treatment ($X^2(1) = 8.9$, $p = 0.0073$) was a significant predictor of the amount of virus detected in a sample, density ($X^2(1) = 3.7$, $p = 0.053$) a marginally significant predictor and drifting ($X^2(1) = 0.53$, $p = 0.47$) was not a significant predictor of the amount of virus detected in a sample. Model estimates are given in Table S4. These results are consistent with the prediction that the likelihood of detecting virus in a sample increases with decreasing numbers of flowers available to a bee.

Table S4 - Model estimates for the likelihood of detecting virus in a bee including a measure of drifting and bee density treated as a continuous variable. Predictor time = the time (days) at which the bee was sampled, treatment = whether the bee was from a non-inoculated or *Crithidia* inoculated colony, density is the average number of flowers available per a bee over the duration of the experiment, and drifting is the percentage of bees in the focal colony that had drifted to or from the SBPV-inoculated colony. Density and drifting measures were standardised across the compartments included in these analyses. Estimates for treatment are for when colonies are '*Crithidia*-inoculated' compared with the reference level of an un-inoculated colony. P-values are not reported for time or treatment alone as their interaction is statistically significant

Predictor	Estimate	SE	Odds ratio	p-value
**time:treatment**	-0.41518	0.13750	0.66	0.007
**time**	0.64541	0.08576	1.9	-
**treatment**	1.00159	0.64069	2.7	-
**density**	-0.94909	0.37886	0.39	0.054
**drifting**	0.14932	0.20113	1.2	0.47
**threshold: 1|2**	**2.6589**	**0.5124**	-	-
**threshold: 2|3**	**5.2171**	**0.6091**	-	-

vii) Initial and final models used to evaluate the transmission of *Crithidia* and SBPV

Table S5 – The effect of bee density on the time taken to detect *Crithidia* in a colony (Senario 2). The initial and final explanatory factors used in the cox-proportional hazards mixed model used in the main manuscript. This model assumes colony 3 in compartment F became infected on day 12 (Scenario 2). 1|Compartment denotes the compartment that a colony was located in was included as a random factor. Density is a factor with the levels high/low. Treatment is a factor with levels recipient/SBPV-inoculated

Model	Density*Treatment	Treatment	Density	1|Compartment
Initial	+<br>$X^2(1) = 0.3011$<br>p = 0.5832	+	+	+
Final		+<br>$X^2(1) = 2.4794$	+<br>$X^2(1) = 0.0327$	+<br>sd = 1.011611

		p = 0.1153	p = 0.8566	

Table S6 – The effect of bee density on the time taken to detect *Crithidia* in a colony (Senario 1). The initial and final explanatory factors used in the cox-proportional hazards mixed model described in the supplementary results. This model assumes colony 3 in compartment F became infected on day 10 (Scenario 1). 1|Compartment denotes the compartment that a colony was located in was included as a random factor. Density is a factor with the levels high/low. Treatment is a factor with levels recipient/SBPV-inoculated

**Model**	**Density*Treatment**	**Density**	**Treatment**	**1|Compartment**
Initial	+ $X^2(1) = 0.4144$ p = 0.5197	+	+	+
Final		+ $X^2(1) = 1.995$ p = 0.1578	+ $X^2(1) = 0.016$ p = 0.8993	+ sd: 1.006709

Table S7 – The effect of bee density on the time taken to detect *Crithidia* in a colony (Senario 2). The initial and final explanatory variables used in the cox-proportional hazards mixed model described in the supplementary results. This model assumes colony 3 in compartment F became infected on day 12 (Scenario 2). 1|Compartment denotes the compartment that a colony was located in was included as a random factor. Density (den) is a standardised covariate representing the number of flowers available to each bee. Drift is a standardised covariate representing the number of bees drifting to/from a *Crithidia*-inoculated colony. Treatment (treat) is a factor with levels recipient/SBPV-inoculated

Model	den*drift*treat	den*drift	den*treat	treat*drift	den	drift	treat	1|Compartment
Initial	+	+	+	+	+	+	+	+
Final					$X^2(1)$ = 1.5091<br>p = 0.2193<br>**HR: 0.72**	$X^2(1)$ = 1.5016<br>p = 0.2204<br>**HR: 0.61**	$X^2(1)$ = 1.7038<br>p = 0.1918<br>**HR(SBPV): 0.50**	+<br>**sd = 0.15544859**

Table S8 – The effect of bee density on the time taken to detect *Crithidia* in a colony (Scenario 1). The initial and final explanatory factors used in the cox-proportional hazards mixed model described in the supplementary results. This model assumes colony 3 in compartment F became infected on day 10 (Scenario 1). 1|Compartment denotes the compartment that a colony was located in was included as a random factor. Density (den) is a standardised covariate representing the number of flowers available to each bee. Drift is a standardised covariate representing the proportion of bees drifting to/from a *Crithidia*-inoculated colony. Treatment (treat) is a factor with levels recipient/SBPV-inoculated

Model	den*drift*treat	den*drift	den*treat	treat*drift	den	drift	treat	1|Compartment
Initial	+	+	+	+	+	+	+	+
final					$X^2(1)$ = 1.77<br>p = 0.1832	$X^2(1)$ = 2.74<br>p = 0.09782	$X^2(1)$ = 1.4767<br>p = 0.2243	+<br>**sd =** 0.0199982703

					**HR: 0.73**	**HR: 0.5698**	**HR(SBPV): 0.54**	

Table S9 – The effect of bee density on the likelihood of virus detection. The initial and final explanatory factors used in the binomial logistic regression mixed model used in the main manuscript. 1|comp/col denotes the compartment that a colony was located in, and colony ID were included as random factors. Density (den) is a factor with the levels high/low. Treatment (treat) is a factor with the levels recipient/*Crithidia*-inoculated. Time (tp) is a covariate representing the time at which samples were taken from a colony

**Model**	**tp:den:treat**	**tp:den**	**tp:treat**	**den:treat**	**tp**	**den**	**treat**	**1|comp**	**1|col**
Initial	+	+	+	+	+	+	+	+	+
Final			+ $X^2(1) = 7.7863$ $p = 0.005264$		+	+ $X^2(1) = 4.0184$ $p = 0.045006$	+	+ **sd = 5.898e-01**	+ **sd = 1.402e-05**

Table S10 – The effect of bee density on the likelihood of virus detection. The initial and final explanatory factors used in the binomial logistic regression mixed model given in the supplementary results. 1|comp/col denotes the compartment that a colony was

located in, and colony ID were included as random factors. Density (den) is a standardised covariate representing the number of flowers available to each bee. Drift is a standardised covariate representing the proportion of bees drifting to/from a SBPV-inoculated colony. Treatment is a factor with levels recipient/*Crithidia*-inoculated. Time (tp) is a covariate representing the time at which samples were taken from a colony

**Model**	**tp:treat: den**	**tp:treat: drift**	**tp:treat**	**tp:den**	**tp:drift**	**treat:den**	**treat:drift**	**tp**	**den**	**drift**	**treat**	**1|comp**	**1|col**
Initial	+	+	+	+	+	+	+	+	+	+	+	+	+
Final			+ $X^2(1)$ = 8.0441 p = 0.004565					+	+ $X^2(1)$ = 4.0143 p = 0.045115	+ $X^2(1)$ = 0.2485 p = 0.618128	+	+ **sd = 5.884e-01**	+ **sd = 3.596e-05**

Table S11 – The effect of bee density on the level of SBPV infection. The initial and final explanatory factors used in the ordinal logistic regression mixed model used in the main manuscript. 1|comp/col denotes the compartment that a colony was located in, and colony ID were included as random factors. Density (den) is a factor with the levels high/low. Treatment (treat) is a factor with the levels recipient/*Crithidia*-inoculated. Time (tp) is a covariate representing the time at which samples were taken from a colony

**Model**	**tp:den:treat**	**tp:den**	**tp:treat**	**den:treat**	**tp**	**den**	**treat**	**1|comp**	**1|col**
Initial	+	+	+	+	+	+	+	+	+

Final			+ $X^2(1) = 8.7279$ p = 0.003134		+	+ $X^2(1) = 4.3584$ p = 0.03683	+	+ **sd = 5.486e-01**	+ **sd = 1.440e-06**

Threshold coefficients for final model:

Estimate Std. Error z value

1|2 1.9985 0.5176 3.861

2|3 4.5287 0.5934 7.632

Table S12 – The effect of bee density on the likelihood of virus detection. The initial and final explanatory factors used in the ordinal logistic regression mixed model given in the supplementary results. 1|comp/col denotes the compartment that a colony was located in, and colony ID were included as random factors. Density (den) is a standardised covariate representing the number of flowers available to each bee. Drift is a standardised covariate representing the proportion of bees drifting to/from a SBPV-inoculated colony. Treatment (treat) is a factor with levels recipient/*Crithidia*-inoculated. Time (tp) is a covariate representing the time at which samples were taken from a colony

**Model**	**tp:treat:den**	**tp:treat:drift**	**tp:treat**	**tp:den**	**tp:drift**	**treat:den**	**treat:drift**	**tp**	**den**	**drift**	**treat**	**1|comp**	**1|col**
Initial	+	+	+	+	+	+	+	+	+	+	+	+	+
Final			+ $X^2(1) =$					+	+ $X^2(1) =$	+ $X^2(1) =$	+	+	+

			8.9365 p = 0.007387						3.7172 p = 0.053854	0.5318 p = 0. 0.465831		**sd: 0.642**	**sd: 0.000**

Threshold coefficients for final model:

Estimate Std. Error z value

1|2 2.6589 0.5124 5.189

2|3 5.2171 0.6091 8.565

viii) The influence of density on SBPV level: Bayesian analyses

The results of our analysis are split into two sections. We first assess whether β is significantly higher than zero. This is sufficient to establish whether density has a significant impact on transmission of SBPV. We then assess how meaningful the impact is, i.e. whether increasing bumblebee density leads to higher SBPV-level observations.

Significance of ß

Under our Bayesian framework, the probability that β is greater than 0 is assessed by identifying the proportion of samples from the posterior distribution of β that are greater than 0. 98.8% of the samples were greater than 0 (Figure S8), meaning that we can reject the null hypothesis that ß = 0 at the 5% level.

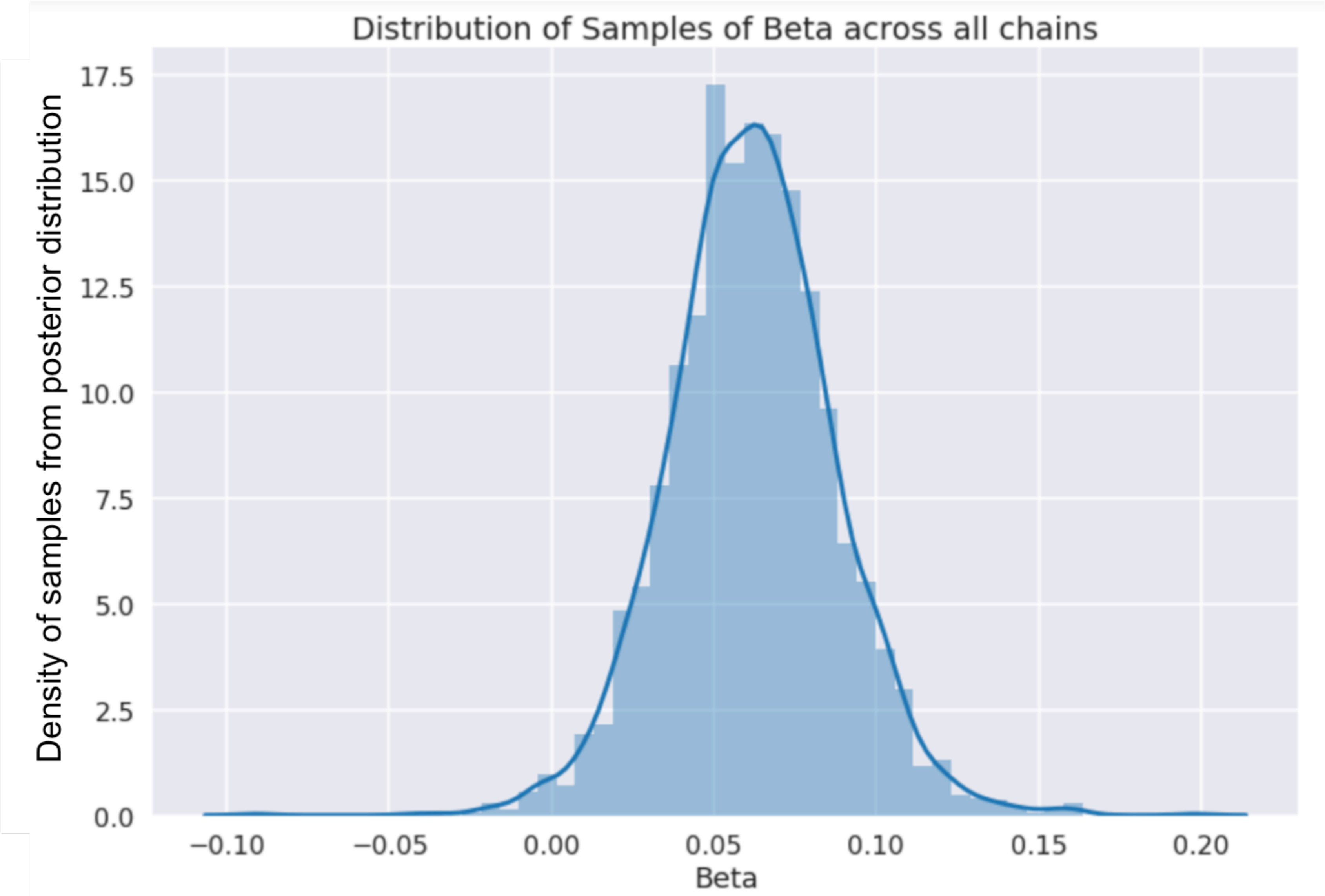

Figure S8 - Distribution of the posterior of Beta (the additive impact of being in a high density compartment on transmission rate)

Magnitude

To determine if the magnitude of ß was biologically relevant, we used the Bayesian framework to "rerun" the experiment. We held all the posterior samples constant and recalculated the predictions for high density compartments using low density compartment transmission rates. Comparing these "rerun" predictions to the model's original predictions for high density compartments allows us to assess the importance of bumblebee density in the transmission of SBPV

The analysis only used the high density compartments as they were the most numerous. To accommodate the very noisy early time points, we started from the third time period and simulated up to the final time period.

We observed a substantial increase in latent SBPV-level using the high density transmission rates rather than the low density transmission rates. The median sample showed a 48% increase in latent SBPV-level, with a lower and upper quartile increase of 32% and 71% respectively. Figure S9 shows the full distribution. This increase in latent SBPV-level corresponds to an increase in the mean observed SBPV-level (ie. a change from level 1 to level 2 etc.) in 47% of samples.

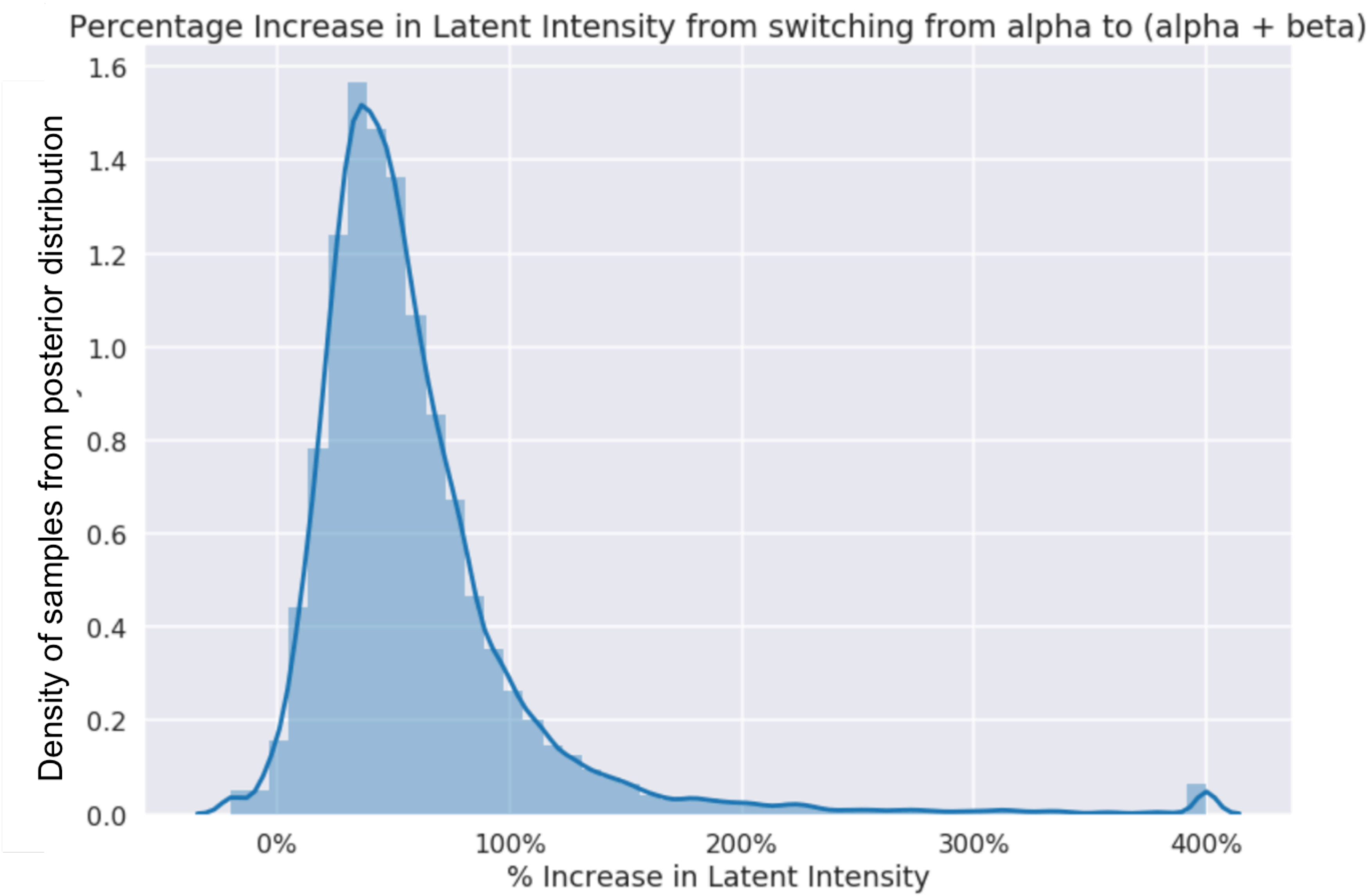

Figure S9 - The increase in latent SBPV-level when high density dynamics rather than low density (ß = 0) dynamics are modeled based on the high density compartments.

Supplementary references

1. Manley R, Boots M, Wilfert L. 2017 Condition - dependent virulence of slow bee paralysis virus in *Bombus terrestris* : are the impacts of honeybee viruses in wild pollinators underestimated ? *Oecologia* **184**, 305–315. (doi:10.1007/s00442-017-3851-2)

2. Wilfert L, Long G, C LH, Schmid-Hempel P, Butlin R, Martin SJ, Boots M. 2016 Deformed wing virus is a recent global epidemic in honeybees driven by Varroa mites. *Science* **351**, 594–597. (doi:10.1126/science.aac9976)

3. Ryabov E V, Wood GR, Fannon JM, Moore JD, Bull JC, Chandler D, Mead A, Burroughs N, Evans DJ. 2014 A virulent strain of Deformed wing virus (DWV) of honeybees (*Apis mellifera*) prevails after *Varroa destructor*-mediated , or in vitro , transmission. *PLoS Pathog.* **10**, e1004230. (doi:10.1371/journal.ppat.1004230)

4. Singh R *et al.* 2010 RNA viruses in Hymenopteran pollinators: evidence of inter-taxa virus transmission via pollen and potential impact on non-Apis Hymenopteran species. *PLoS One* **5**, e14357. (doi:10.1371/journal.pone.0014357)

5. Wickham H. 2016 *ggplot2: elegant graphics for data analysis*. New York: Springer-Verlag.

6. Lüdecke D. 2018 sjPlot: Data Visualization for Statistics in Social Science. R package version 2.4.1.9000.

7. R Core Team. 2017 R: A language and environment for statistical computing.

8. Harrell Jr FE. 2018 rms: Regression Modeling Strategies. R package version 5.1-2.

9. Fox J, Weisberg S. 2011 *An {R} Companion to Applied Regression*. 2nd edn. Thousand Oaks CA: Sage.

10. Therneau TM, Grambsch PM. 2000 *Modeling Survival Data: Extending the Cox Mode*. New York: Springer.

11. Therneau TM. 2015 A package for survival analysis in S.

12. Bates D, Mächler M, Bolker B, Walker S. 2015 Fitting linear mixed-effects models using lme4. *J. Stat. Softw.* **67**, 1–48.

(doi:10.18637/jss.v067.i01)

13. Christensen RHB. 2018 ordinal - Regression models for ordinal data. R package version 2018.4-19.
